# New Engineered-Botulinum Toxins Inhibit the Release of Pain-Related Mediators

**DOI:** 10.3390/ijms21010262

**Published:** 2019-12-30

**Authors:** Minhong Tang, Jianghui Meng, Jiafu Wang

**Affiliations:** School of Biotechnology, Faculty of Science and Health, Dublin City University, Collins Avenue, Glasnevin, Dublin 9, Ireland; minhong.tang3@mail.dcu.ie

**Keywords:** cytokine, neuropeptides, neurotoxin, therapeutics, targeting, protein conjugation

## Abstract

Targeted delivery of potent inhibitor of cytokine/pain-mediator into inflammatory or pain-sensing cells is a promising avenue for treating chronic pain, a world-wide major healthcare burden. An unmet need exists for a specific and effective delivery strategy. Herein, we describe a new approach using sortase to site-specifically ligate a non-toxic botulinum neurotoxin D (BoNT/D) core-therapeutic (synaptobrevin-cleaving protease and translocation domains) to cell-specific targeting ligands. An engineered core-therapeutic was efficiently ligated to IL-1β ligand within minutes. The resultant conjugate specifically entered into cultured murine primary macrophages, cleaved synaptobrevin 3 and inhibited LPS/IFN-γ evoked IL-6 release. Likewise, a CGRP receptor antagonist ligand delivered BoNT/D protease into sensory neurons and inhibited K^+^-evoked substance P release. As cytokines and neuropeptides are major regulators of inflammation and pain, blocking their release by novel engineered inhibitors highlights their therapeutic potential. Our report describes a new and widely-applicable strategy for the production of targeted bio-therapeutics for numerous chronic diseases.

## 1. Introduction

Chronic pain, including rheumatoid arthritis (RA), is still a major medical challenge, with a high number of people suffering from persistent pain worldwide. Currently, three main types of medicine are widely used to treat chronic pain, including nonsteroidal anti-inflammatory drugs (NSAIDs) and opioids with adjuvants such as antidepressants and anticonvulsants. Unfortunately, these medications are largely ineffective or unsuitable for many sufferers of chronic pain, either due to their short half-lives or various unacceptable adverse side effects, including addiction and overdose [1]. Thus, a pressing unmet need exists for persistently-acting and non-addictive medication for chronic pain.

Toward this end, botulinum neurotoxins (BoNTs) have been proposed to hold significant potential as local treatments for chronic pain. Clinical research demonstrated that after treatment with BoNT/A complex (BOTOX^®^) to migraine patients, their headache pain symptoms were significantly alleviated, and the frequency and duration of migraines were also reduced [2]. There are seven serotypes of BoNTs present in nature, termed BoNT/A to BoNT/G, each with a molecular weight of approximately 150 kDa. Sequence comparison combined with employing serotype-specific antibodies has revealed dozens of BoNT subtypes (BoNT/A1, BoNT/A2, BoNT/B2, etc.) and mosaic toxins (e.g., BoNT/CD, BoNT/DC, BoNT/FA(H)) [3,4,5,6,7,8]. Moreover, using bioinfomatics analysis approach, a novel serotype BoNT/X was discovered [9]. BoNT serotypes are similar in both general function and structure, consisting of a ≈50 kDa N-terminal protease light chain domain (LC) and a ≈100 kDa C-terminal heavy chain domain (HC), linked via a disulphide bond. Most of BoNTs target neurons by binding to gangliosides and the synaptic vesicle protein 2 (serotypes: /A, /D–/F) or synaptotagmin (/B and /G) via the C-terminal half of HC (H_C_) (in [10]). Once BoNTs get internalised into the neurons, the N-terminal half of HC (H_N_) forms a channel on the synaptic vesicle and/or endosomal membrane, allowing for translocation of the attached LC to the cytosol [11]. This active LC protease can then cleave and inactivate soluble *N*-ethylmaleimide-sensitive factor attachment protein receptors (SNAREs). Synaptosomal-associated protein 25 kDa (SNAP-25) is cleaved by LC/A,/C1 and/E whereas synaptobrevin, also known as vesicle associated membrane protein (VAMP) isoform 1, 2 and 3 are truncated by LC/B,/D,/F and/G. BoNT/C1 additionally cleaves syntaxin 1. Uniquely, BoNT/X not only truncates VAMP 1, 2 and 3, but also non-traditional SNAREs, such as VAMP4, VAMP5 and Ykt6 [9], possibly extending the therapeutic uses of BoNTs. Cleavage of these SNAREs results in the disruption of the vesicle fusion and blockade of transmitter release (reviewed in [10]). BoNT serotypes/A and /B complex forms have been widely used for treating hyper-excitability disorders of cholinergically innervated muscles or glands. In 2010, BOTOX^®^ was approved by the FDA to treat chronic migraines [12]. As an analgesic agent, it has the advantage of being non-additive and of having a long duration of action, due to persistent cleavage of SNAP-25 [12,13]. However, BoNTs can produce unwanted secondary effects (e.g., muscle paralysis), often due to diffusion of the toxin from the injection site to non-targeted structures [14]. To avoid the unwanted muscle paralysis side effect associated with the treatment of chronic pain, improvement of BoNT is desired so that it will selectively and specifically target BoNT SNARE-cleaving protease into inflammatory cells and/or sensory neurons rather than motor neurons.

RA is a common autoimmune disorder characterized by chronic joint inflammation, swelling and pain. Macrophages are known to play an important role in the pathogenesis and progression of RA, primarily through the secretion of various inflammatory mediators, resulting in cartilage and bone destruction [15]. The most important pro-inflammatory cytokines which play an essential role in the development of RA are tumor necrosis factor-α (TNFα), interleukin 1β (IL-1β) and IL-6 [16,17,18]. TNFα and IL-6 sensitize joint nociceptors to mechanical stimulation, and thus, directly contribute to mechanical hyperalgesia [19]. It is known that macrophages require VAMP3 to secrete cytokines [20,21]. Thus, targeted delivery of VAMP-cleaving protease into macrophages could offer a novel means for potential treatment of RA disease. Hence a site-specific, robust, reliable and efficient strategy for modification of BoNTs is highly desirable for generating retargeted BoNTs’ proteases. In order to meet this demand, we exploited the sortase-mediated protein ligation technique to make functional targeted BoNT based fusion proteins by stitching BoNT protease to the cell-specific targeting ligands. *Staphylococcus aureus sortase* A, a thiol transpeptidase, exists in many Gram-positive bacteria and is responsible for covalent anchoring of cell surface proteins to bacterial cell walls [22]. Under physiological reaction conditions, proteins with an exposed LPXTG motif (X: any residue) can be specifically ligated by sortase A to an aminoglycine protein/peptide via an amide bond.

Using general molecular biology techniques, a short, non-structural linker followed by LPETG motif was attached to the C-terminal of long-acting BoNT/D core-therapeutic consisting of LC and H_N_ domains lacking the neuronal binding domain H_C_ (/DΔH_C_). The resultant protein /DΔH_C_-CS (CS refers to the C-terminal sortase motif) was expressed in *Escherichia coli* and purified with retention of its full VAMP cleaving protease activity. This protein was ligated to a recombinantly produced interleukin 1β (IL-1β) or a synthesized calcitonin gene-related peptide (CGRP) receptor antagonist (CGRP_8–37_) within minutes via a sortase-catalyzed reaction to produce the retargeted BoNT/D based therapeutic candidates: /DIL-1β and /D-CGRP_8–37,_ respectively. As macrophages express the IL-1 receptor [23,24] and a fraction of small to medium-sized dorsal root ganglion neurons (DRGs) express the CGRP receptor [25], the above mentioned ligated ligands successfully delivered the BoNT/D core-therapeutic into either cultured macrophages or DRGs. This results in inhibiting the release of inflammatory cytokines or pain transmitter peptides (substance P). Thus, our findings indicate that these retargeted BoNT/D-based therapeutics possess anti-inflammatory and/or anti-nociceptive capabilities. Moreover, due to the rapid, reliable and robust nature of the method described herein, we believe that this retargeting strategy will prove to be a valuable and widely-applicable tool for the development of future BoNT-based therapeutics.

## 2. Results

### 2.1. BoNT/D Core-Therapeutic with Sortase A Recognition Motif Was Expressed and Purified with Good Yield and Purity

**Figure 1:**
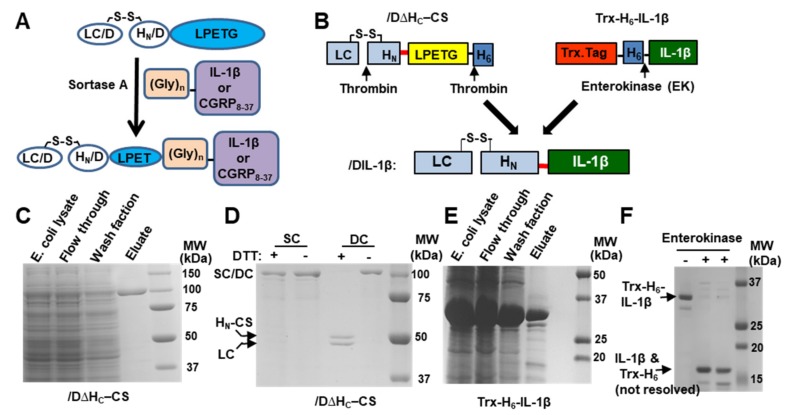
Protein engineering BoNT/D core-therapeutic and targeting ligand. (**A**) Schematic of the sortase A-mediated conjugation strategy. Sortase A can ligate recombinant (Gly)_5_-IL-1β or attach (Gly)_3_-CGRP_8-37_ to the C-terminal of LC.H_N_/D via recognition of the LPETG motif and cleavage of the bond between T and G. (**B**) Illustration of protein engineering /DIL-1β via ligation of Gly_5_-IL-1β to /D∆H_C_-CS by sortase A. /D∆H_C_–CS depicts BoNT/D core-therapeutic with a C-terminal sortase and thrombin recognition motifs (see Methods). Trx: thioredoxin; H_6_: His_6_ tag; CS, C-terminal sortase motif. The red bold bar between H_N_ and IL-1β denotes a short peptide sequence consisting of LPETG and non-structural linkers located on each end (see Methods). (**C**) /D∆H_C_-CS was expressed in *Escherichia coli* and purified by IMAC. Aliquots from IMAC were analyzed by SDS-PAGE followed by Coomassie staining. (**D**) /D∆H_C_-CS SC and thrombin-nicked di-chain (DC) were subjected to SDS-PAGE followed by Coomassie staining. (**E**) Purification of Trx-H_6_-IL-1β fusion protein by IMAC. (**F**) SDS-PAGE analysis of Trx-H_6_-IL-1β protein with or without enterokinase treatment. IL-1β and Trx-H_6_ have similar molecular weights (≈17 kDa).

The sortase A-mediated conjugation strategy was chosen to re-direct BoNT/D core-therapeutic into the target cells. This method allows for efficient ligation of a targeting ligand (peptides or proteins with or without modification) to the core-therapeutic (Figure 1A). First, a synthetic gene fragment encoding LC.H_N_ of BoNT/D (denoted /DΔH_C_), with a codon optimized for *E. coli* expression, was inserted into the pET29a vector. Note that this synthetic gene contains a thrombin recognition consensus site at the loop region between the LC and H_N_ domains, allowing for precise nicking. Subsequently, a short nucleotide sequence encoding a non-structural linker and a sortase A recognition motif (LPETG) followed by a thrombin recognition sequence was inserted between the 3′end of H_N_/D gene and vector nucleotides encoding a C-terminal His_6_ tag. This generated a construct, encoding /D∆H_C_-CS (CS refers to the C-terminal sortase motif) (Figure 1B). After transformation of the resultant plasmid into *E. coli* BL21 DE3, /D∆H_C_-CS was expressed in *E. coli* using an auto-induction medium and successfully purified by immobilised metal ion affinity chromatography (IMAC) with a yield of (≈4 mg/L of culture). /D∆H_C_-CS was expressed and purified as the single-chain (SC) form with the predicted molecular weight (≈100 kDa) (Figure 1C). The purified/D∆H_C_-CS SC was then nicked into the di-chain (DC) form by thrombin. This was examined by SDS-PAGE in the presence or absence of a reducing agent, dithiothreitol (DTT). The nicked sample remained a single band in the absence of the reducing agent, and its constituents (LC and H_N_-CS) were only separated in the presence of DTT, confirming that the disulphide interchain was successfully formed in the *E.coli* (Figure 1D).

### 2.2. Protein Engineering Inflammatory Cell Targeting Ligand and Its Efficient Conjugation to BoNT/D Core Therapeutic via Sortase Enzyme

To target BoNT/D core-therapeutic into inflammatory cells, we selected the IL-1β as a targeting ligand because IL-1 receptors are expressed on certain inflammatory cells, including macrophages, synoviocytes, etc. [23,24]. We inserted the synthetic gene encoding Gly_5_-IL-1β protein into a bacterial expression vector pET32b immediately after the enterokinase cleavage site to create a construct, encoding thioredoxin-His_6_-Gly_5_-IL-1β protein (Figure 1B, abbreviated as Trx-H_6_-IL-1β). This strategy allows for enterokinase-mediated removal of the thioredoxin-His_6_ tags from the Gly_5_-IL-1β, and subsequent ligation of Gly_5_-IL-1β to the BoNT/D core-therapeutic (Figure 1B). Similarly, Trx-H_6_-IL-1β fusion protein was expressed in BL21.DE3 and purified by IMAC with a yield of (≈60 mg/L of culture). Purified fusion protein was visualized on the Coomassie stained SDS-PAGE gel with *Mr* ≈ 34 kDa (Figure 1E). Incubation of fusion protein with enterokinase released Gly_5_-IL-1β from Trx-His_6_ tags, yielding the predicted size of *Mr* ≈ 17 kDa, corresponding to the removal of Trx-His_6_ tag (abbreviated as Trx-H_6_) (Figure 1F). As IL-1β and Trx-H_6_ are of similar molecular weight, they were not resolved by SDS-PAGE (Figure 1F).

**Figure 2:**
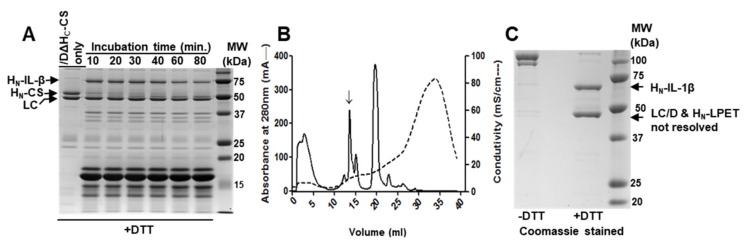
Production of /DIL-1β. (**A**) D∆H_C_-CS was incubated with sortase A, and enterokinase cleaved Trx-H_6_-IL-1β mixture at 37 °C. Samples were taken at different time points and subjected to SDS-PAGE in the presence of 50 mM DTT followed by Coomassie staining. (**B**) Anion-exchange chromatography was used to separate /DIL-1β conjugate from the unconjugated product and contaminants. Arrow indicates the eluted peak for /DIL-1β. Solid line and broken line represent the absorbance and conductivity, respectively. (**C**) Pooled /DIL-1β product from **B** was subjected to SDS-PAGE followed by Coomassie staining.

Incubation of enterokinase cleaved product mixture with /DΔH_C_-CS in the optimized reaction condition; sortase A rapidly ligated IL-1β to /DΔH_C_-CS within 10 min to yield /DIL-1β conjugated protein. Further incubation did not result in higher yield (Figure 2A). /DIL-1β product was further purified by anion-exchange chromatography (AEX) to remove unbound IL-1β substrate and contaminant present (Figure 2B). The final product was analyzed by SDS-PAGE in the absence or presence of DTT. The majority of /DΔH_C_-CS was conjugated to IL-1β (Figure 2C, top band in the left lane) with mobility slower than the unconjugated form (Figure 2C, lower band in the left lane). In the presence of DTT, the LC/D and H_N_/D-IL-1β were separated (Figure 2C), confirming that the inter-chain disulphide bond was not affected by the reaction. The LC/D and sortase A-cleaved H_N_-LPET in the un-conjugated protein were also segmented in the presence of DTT but not resolved in the SDS-PAGE due to having similar molecular weights (Figure 2C).

### 2.3. /DIL-1β Conjugate Exerts Receptor Binding and SNARE-Cleaving Biological Activities

**Figure 3:**
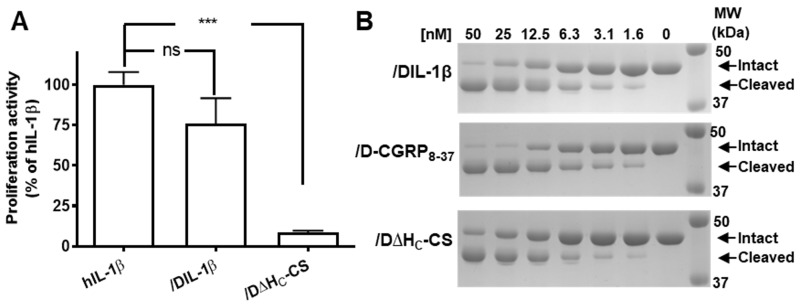
**/**DIL-1β retains biological activities of its ligand and protease. (**A**) Bar chart showing RAW cell proliferation activity of /DIL-1β and /D∆H_C_-CS relative to commercial human IL-1β. Data plotted are mean ± S.E.M. (*n* = 3). ns: non-significant, *** *p* < 0.001. (**B**) /DIL-1β, /D-CGRP_8-37_ and /D∆H_C_-CS have similar protease activities regarding cleaving a recombinant GFP-VAMP2_2-94_-His_6_ substrate.

Next, it was important to confirm that, following ligation to /D∆H_C_-CS, the IL-1β component remained biologically active and could bind its cognate receptor. We used a cell proliferation method to assess its activity in RAW 264.7 macrophage cells. /DIL-1β application was found to promote cellular proliferation, showing only a slight, but not significant decrease of activity compared with commercial human IL-1β (Figure 3A). This confirms that the ligated product retains the bio-activity of its ligand. As expected, /DΔH_C_-CS (the non-targeted control protein) did not induce cell proliferation (Figure 3A). Proteolytic activity was then investigated using a recombinant model substrate GFP-VAMP2_(2-94)_-His_6_. The /DIL-1β conjugate was found to possess similar protease activity to the non-targeted control protein (/D∆H_C_-CS) (Figure 3B). Thus, ligation of the targeting ligand to /D∆H_C_-CS did not result in loss of ligand- or protease-mediated activity.

### 2.4. IL-1β Successfully Delivered BoNT/D VAMP-Cleaving Protease into RAW264.7 Cells and Primary Peritoneal Macrophages Resulting in Inhibition of IL-6 Release

**Figure 4:**
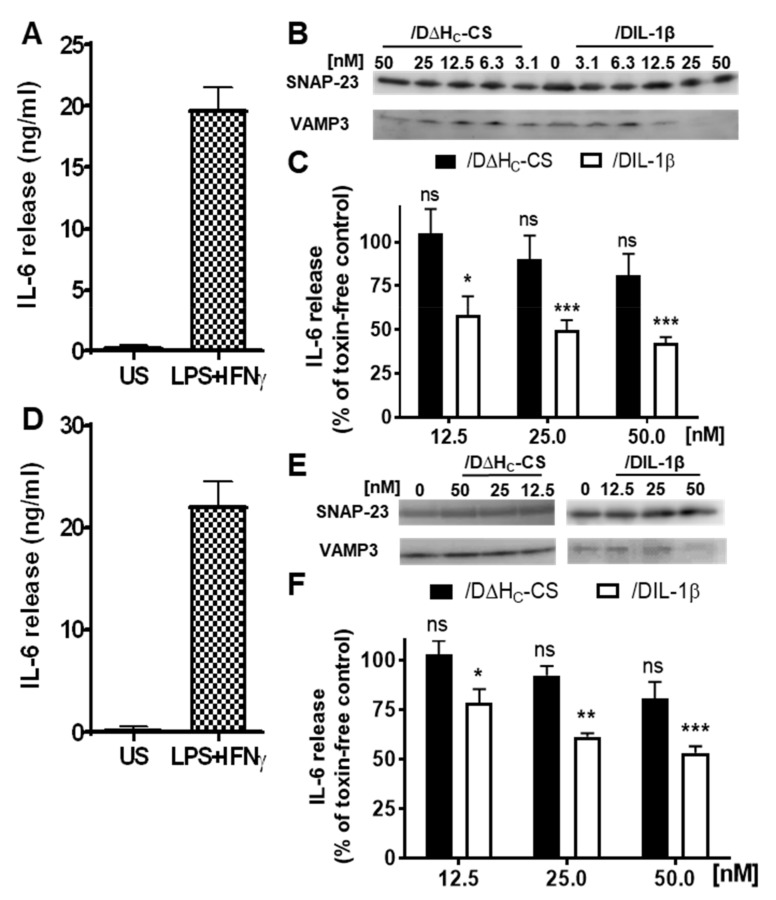
**/**DIL-1β entered the cultured macrophages, cleaved VAMP3 and inhibited evoked IL-6 release, unlike the untargeted control. (**A**) Treatment of RAW 264.7 cells with LPS and IFNγ for 42 h induced substantial IL-6 release in comparison to unstimulated (US) cells. (**B**) Cleavage of VAMP 3 in RAW 264.7 cells by treated proteins was probed by Western blotting using an antibody against VAMP1/2/3 forms. Note that RAW cells predominately express the VAMP3 isoform (Figure S1). It is worth noting that the antibody used only recognises the intact but not the BoNT/D-protease-cleaved form. SNAP-23 was probed as an internal loading control. (**C**) /DIL-1β and /D∆H_C_-CS inhibited LPS and IFNγ evoked IL-6 release from the cultured RAW 264.7 cells to different extents. (**D**–**F**) Experiments were repeated as for (**A**–**C**), except primary mouse macrophage cells were used. Data plotted are mean ± S.E.M. (*n* = 3). Significance between /DIL-1β or /D∆H_C_-CS treated cells and toxin non-treated control cells is indicated as follows: non-significant (ns), *p* > 0.05; * *p* < 0.05; ** *p* <0.01 and *** *p* < 0.001.

As mentioned in the introduction, macrophages play an important role in the pathogenesis of chronic inflammatory diseases, including RA. Macrophages also express the IL-1 receptor and certain SNARE proteins, especially SNAP-23 and VAMP3 [23,24,26]. Cultured RAW264.7 macrophages were treated with or without /DIL-1β for 6 h before stimulation of cytokine release with interferon gamma (IFNγ) and lipopolysaccharides (LPS) for 42 h. Upon stimulation with IFNγ and LPS, cultured macrophages secreted a large quantity of the pro-inflammatory cytokine IL-6 (Figure 4A). Importantly, /DIL-1β pretreatment resulted in both VAMP3 cleavage (Figure 4B and Figure S2) and a striking inhibition of IL-6 release (Figure 4C). In contrast, the non-targeted control protein /D∆H_C_-CS failed to achieve comparable VAMP3 cleavage (Figure 4B and Figure S2), and even at the highest concentration tested, only caused a slight inhibition of IL-6 release (Figure 4C). This experiment was then repeated on primary macrophages isolated from mouse peritoneal cavity. Similar to RAW264.7 macrophages, stimulation with IFNγ and LPS promoted IL-6 release from cultured primary macrophages (Figure 4D). Reassuringly, /DIL-1β entered primary macrophages, truncated VAMP3 (Figure 4E and Figure S2) and attenuated IL-6 release more potently than the control protein (Figure 4F). Note that neither the targeted nor non-targeted control toxins affected cell viability (Figure S3). Thus, our results confirm that fused IL-1β can deliver BoNT/D protease specifically into macrophages.

### 2.5. Conjugating a CGRP Antagonist to /DΔH_C_-CS Targets Sensory Neurons and Inhibits Pain-Peptide Release

**Figure 5:**
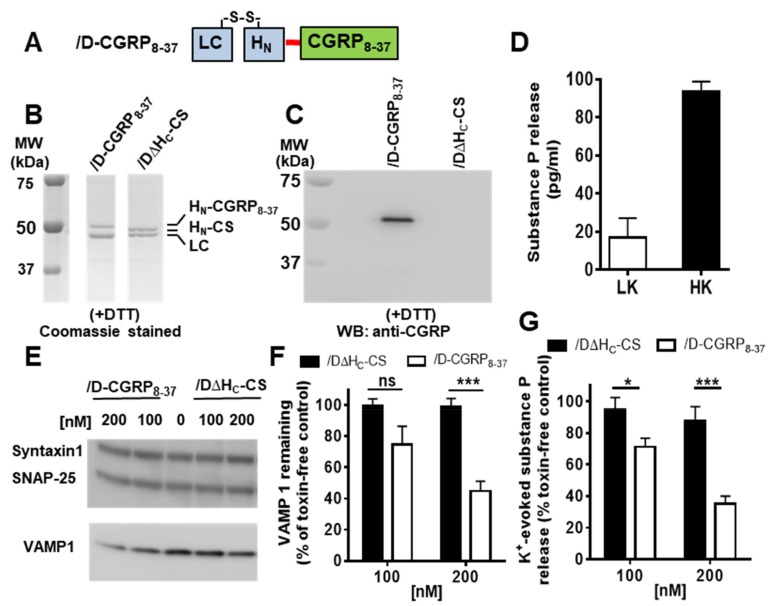
Conjugation of CGRP_8-37_ to /D∆H_C_-CS yielded /D-CGRP_8-37_ which cleaved VAMP1 and inhibited depolarization evoked substance P release from cultured DRGs. (**A**) Schematic of /D-CGRP_8-37_. (**B**, **C**) DTT reduced /D-CGRP_8-37_ and /D∆H_C_-CS samples were analyzed by SDS-PAGE followed by Coomassie staining (**B**) or by Western blotting with a rabbit anti-CGRP antibody (1:10,000) (**C**). Uncropped original images are shown in Figure S4. (**D**) Depolarization of cultured DRGs stimulated the release of substance P approximately 5-fold over basal release (LK, low potassium; HK, high potassium). (**E**) Representative Western blots showing the cleavage of VAMP1 by /D-CGRP_8-37_ compared to /D∆H_C_-CS. Syntaxin1 and SNAP-25 were probed as loading control. (**F**) Intact VAMP1 remaining after incubating with 100 nM or 200 nM of either protein was calculated by expressing the ratio (intensity of VAMP1/the corresponding internal loading control (Syntaxin1)) as a percentage of the toxin free control sample. (**G**) /D-CGRP_8-37_ but not/D∆H_C_-CS attenuated the K^+^-evoked substance P release when compared to the toxin-free sample. Data plotted in (**D**,**F**,**G**) are mean ± S.E.M. (*n* = 3). ns: non-significant, * *p* < 0.05 and *** *p* < 0.001.

Our results have confirmed that sortase A enzyme can efficiently ligate the small recombinant protein to the BoNT/D core therapeutic with retention of the biological activities of ligand and SNARE-cleaving protease. To target /DΔH_C_-CS into sensory neurons for potential pain relief, we exploited a CGRP antagonist as a targeting ligand because a subset of sensory neurons are known to express CGRP receptors [25]. A truncated CGRP peptide, CGRP_8-37_, binds to CGRP receptor and antagonizes CGRP activity [27]. After 30 min incubation of a synthesized Gly_3_-CGRP_8-37_ peptide with /DΔH_C_-CS and sortase A, /D∆H_C_-CS was ligated to CGRP_8-37_, yielding /D-CGRP_8-37_ conjugate as demonstrated by a shift towards higher molecular size in the SDS-PAGE gel (Figure 5A,B). Anti-CGRP antibody recognized the /D-CGRP_8-37_ sample but not the /DΔH_C_-CS (Figure 5C), further confirming CGRP_8-37_ successfully conjugated to /D∆H_C_-CS. Cultured rat DRGs were incubated with or without /D-CGRP_8-37_ for 24 h before stimulation with 60 mM KCl for 30 min. Depolarization of DRGs stimulated the release of substance P approximately 5-fold over basal release (Figure 5D). Treatment with /D-CGRP_8-37_ led to a significant cleavage of VAMP1, especially at 200 nM (Figure 5E,F), resulting in a substantial reduction of potassium evoked substance P release (Figure 5G). In contrast, non-targeted control protein failed to cleave VAMP1 and inhibit substance P release (Figure 5E–G). Thus, our data confirmed that, following successful addition of the CGRP_8-37_ peptide, active BoNT/D protease can be effectively targeted into sensory neurons.

## 3. Discussion

Here we have demonstrated an efficient and robust method for ligation of recombinantly produced BoNT core-therapeutics to ligands (either recombinant protein or synthesized peptide), targeting the delivery of SNARE-cleaving protease into specific cell types. Our strategy is dependent on removal of the BoNT/D neuronal receptor binding domain, followed by sortase A-mediated conjugation of the targeting ligand. Functional analyses show that our targeted BoNT proteases bind and enter rodent macrophages or sensory neurons, cleave SNAREs and block evoked release of cytokines or pain peptides, thereby possessing anti-inflammatory and/or anti-nociceptive potential.

Production of active botulinum neurotoxins requires strict safety containment not just for the process itself but also for the staff and the laboratory environment. Very few laboratories have the authority to produce these exceptionally potent molecules. This could be achieved by separately expressing two inactive BoNT segments followed by their assembly in reaction tubes in the presence of sortase A. This technology could also be useful for preparing other toxin molecules, such as ricin, diphtheria toxin or *Pseudomonas* exotoxin. Thus, sortase A mediated ligation technology for producing therapeutic molecules or research tools may offer multiple advantages in addition to no chemical manipulation or activation of the *N*-peptides and *C*-peptides: (i) to overcome the safety issues due to toxicity of expressed protein in host; (ii) to attach active targeting proteins which require posttranslational-modifications; (iii) to incorporate of non-native peptides and non-peptidic molecules into proteins; (iv) to attach small fluorescent probes to the targeting peptides during synthesis to track the molecules in the targeted cells. Previously, researchers have used protein fusion, chemical conjugation or protein stapling by exploiting tight interaction among SNAP-25/VAMP2/Syntaxin 1 fragment for protein engineering potent chimeric neurotoxins or retargeted BoNTs [9,28,29,30,31,32,33,34,35,36,37,38,39,40,41,42,43,44,45].

We chose sortase A mediated technology to engineer BoNT-based targeted therapeutic candidates as potential treatments for RA and/or neuropathic pain. RA poses a major health and economic burden. The pathogenesis of arthritis is not yet fully understood. Nevertheless, the abundance and activation of macrophages in the inflamed synovial membrane/pannus significantly correlate with the severity of RA [46]. No arthritis cure exists at present. The advent of the biotherapeutics has now radically changed the approach to treatment, because these can relieve pain in some patients by blocking the effects of endogenous pain mediators, thereby exerting analgesic and anti-inflammatory actions. Administering monoclonal antibodies against TNFα or IL-6 or their receptors has proven beneficial in clinical therapy of patients with RA [47,48]. Although these bio-therapeutics have yielded encouraging results, their high price, numerous adverse reactions and diminishing efficacy over time emphasize the urgent need for improved versions. Yeh et al. adopted the antibody-mediated delivery method to target BoNT/B protease into macrophages via the Fc and complement receptor-mediated endocytosis pathway. A BoNT/B and an anti-BoNT/B antibody mixture entered macrophages and blocked the release of cytokines [49]. Herein, exploiting the presence of IL-1R on the macrophage, we produced the recombinant IL-1β and used it as a targeting ligand to bind macrophages for subsequent cytosolic delivery of BoNT/D VAMP-cleaving protease, a step requiring the endosomal membrane channel formation via the H_N_ domain. Our results clearly demonstrate that the IL-1β-fused BoNT/D core-therapeutic molecule can enter cultured macrophage cell lines as well as murine primary macrophages because of the observed cleavage of its intracellular targets and eventual blockade of cytokine exocytosis, in contrast to non-targeted control. The current study is also in accordance with our earlier findings and other groups’ data that VAMP3 is required for IL-6 release [26,50]. Previous report has claimed that a BoNT lacking the binding domain was able to enter cultured cells and cleave intracellular targets, albeit at high concentrations [51]. We observed some cleavage of VAMP3 and inhibition of IL-6 release in our non-targeted control toxin-treated samples. Due to the phagocytic nature of macrophage cells, we speculate that this effect may be mediated by non-specific endocytosis of the control toxin. That said, as addition of the IL-1β ligand allowed for specific targeting of the BoNT/D protease into IL-1R-expressing macrophages and enhancing the efficacy of this modified BoNT/D, we believe that our recombinant targeted toxin would be a potent and effective treatment for RA.

Peripheral nerve injury results in the release of several pain-peptides from primary nociceptive afferents. These include substance P and CGRP, which are involved in nociceptive processing in both the peripheral and the central nervous systems [52]. They also regulate various functions of cells, including macrophages, synoviocytes and T cells and augment the production of pro-inflammatory cytokines, prostaglandin E2 or collagenase [53,54,55]. Thus, developing new therapeutics either inhibiting the release of these two neuropeptides or blocking their receptor activation is fully warranted [56,57]. CGRP_8–37_ has been demonstrated to inhibit vasodilation and neurogenic inflammation in animal models due to blocking binding of endogenous CGRP to CGRP receptors. However, its clinical effectiveness is limited due to its short half-life [27]. Interestingly, Dragon’s blood, which is a traditional Chinese medicine, can control inflammation and relieve pain symptoms by inhibiting the secretion of substance P [58]. Moreover, local injection of BoNT/A complexes or recombinantly engineered BoNTs attenuated the neuropathic pain in rodent models [59,60,61], perhaps by blocking pain-peptide release from peripheral nociceptive fibers. In this study, we selected CGRP_8–37_ as a targeting ligand for selective delivery of BoNT/D protease into sensory neurons with the aim of attenuating pain-peptide release. /D-CGRP_8–37_ gave much more pronounced cleavage of VAMP1 and inhibition of depolarization-evoked substance P release from cultured mouse DRGs than non-targeted control proteins, highlighting the specific uptake of BoNT/D protease through the ligated CGRP_8–37_ ligand. Although inhibition of substance P release by/D-CGRP_8–37_ was incomplete, this might be due to only a subpopulation of DRGs co-expressing CGRP receptor and neuropeptide [25]. We also produced CGRP_8–37_ fused BoNT/D core therapeutic fusion protein in *E.coli*, which proved a lack of significant improvement in SNARE cleavage and inhibition of neuropeptides release from DRGs when compared to non-targeted control protein. It seems that C-terminal amidation of CGRP_8–37_ is essential for targeting efficacy. This hypothesis accords with an earlier finding that replacement of the C-terminal amide in CGRP peptide with a carboxyl results in substantial reduction in human CGRP receptor 1 affinity [62]. This also proves the advantages of using sortase A mediated strategy by independently producing two active therapeutic components. An earlier study reported that an intracisternal injection of a chemically conjugated substance P-LC/A decreased thermal hyperalgesia in a mouse model of Taxol induced neuropathic pain [63]. A recent study reported that intrathecal administration of a stapled LC.H_N_/A-substance P conjugate induced long-term reduction of inflammatory and neuropathic pain sensitivity in mice [39]. We also tried to use synthesized substance P as a targeting ligand and found substance P is slightly less effective than CGRP_8–37_ at delivering BoNT/D protease into DRGs, as reflected by the extent of VAMP1 cleavage (Figure S4).

The targeted delivery of a SNARE protease to specific neuronal subtypes or non-neuronal cells represents a revolutionary approach for pain relief. Overall, we have described a method for protein engineering BoNT-derived targeted molecules—potential therapeutic candidates for treating RA or neuropathic pain. Inhibiting the release of pro-inflammatory cytokines or major pain neuropeptides by these reagents encourages further studies on effects of these candidates on animal models of inflammatory and neuropathic pain.

## 4. Materials and Methods

### 4.1. Materials

Talon cobalt resin was supplied by Clontech Laboratories, Inc. (Mountain View, CA, USA). Resource Q and PD-10 columns were bought from GE Healthcare (Dublin, Ireland). Thrombin enzyme, vectors and the enhanced chemiluminescence reagent (ECL) were ordered from Merck Millipore (Cork, Ireland). Enterokinase and restriction enzymes were bought from New England Biolabs (Dublin, Ireland). RAW 264.7 cell line murine macrophage and rabbit anti-rat CGRP polyclonal antibody were ordered from Sigma-Aldrich (Arklow, Ireland). Mouse nerve growth factor (NGF-2.5S) was bought from Alomone Labs (Jerusalem, Israel). Zeba Spin Desalting Columns, B-27 Supplement and Collagenase I were supplied by Life Technologies Ltd. (Paisley, UK). Dispase II was bought from Roche Products Ltd. (Dublin, Ireland). Human IL-1β and mouse IL-6 Duo-set ELISA kits were obtained from R&D Systems Eire (Abingdon, UK). SP enzyme immunoassay (EIA) kit from Cayman Chemical was supplied by Bertin Bioreagent (York, UK). Rabbit anti-rat SP polyclonal antibody was ordered from Enzo Life Sciences LTD (Exeter, UK). Synaptic Systems GmbH (Goettingen, Gremany) supplied rabbit VAMP1/2/3 polyclonal antibody and VAMP1 monoclonal antibody. Horseradish peroxidase-conjugated donkey anti-rabbit and anti-mouse secondary antibodies were purchased from Jackson ImmunoResearch (Suffolk, UK). Click-iT^®^ EdU microplate assay kit and Bolt 12% Bis-Tris gels were purchased from Bio-Sciences (Dun Laoghaire, Ireland). Rat Gly_3_-CGRP_8-37_ and Gly_3_-substance P peptides with C-terminal amide modifications were synthesized by LifeTein, LLC (Somerset, NJ, USA). All other reagents and chemicals were ordered from Sigma-Aldrich (Arklow, Ireland). Gene synthesis and DNA sequencing were serviced by Eurofins Genomics (Ebersberg, Germany). pet30b-7M SrtA encoding hepta-mutant *Staphylococcus aureus* sortase A was a gift from Hidde Ploegh (Addgene plasmid number 51141).

### 4.2. Animal Ethics Statement

Sprague Dawley rat pups and CD1 mice were bred in the Bio Resources Unit, Dublin City University (DCU). All of the procedures involving living animals were approved by the Dublin City University Research Ethics Committee and by the Health Products Regulatory Authority under Directive 2010/63/EU and the European Union (Protection of Animals Used for Scientific Purposes) Regulations 2012 (S.I. number 543 of 2012).

### 4.3. Production of/D∆H_C_-CS, Trx-H_6_-IL-1β and Sortase A

The experiment involving production of recombinant protein and their treatment with cells was approved by the Dublin City University (DCU) biological Safety Committee and the Environmental Protection Agency of Ireland. The synthetic gene encoding /D∆H_C_ was cloned into the NdeI and SacI sites in the pET29a vector. A short nucleotide encoding a non-structural linker 2× (Gly_4_Ser) and **LPETG**GGGSGGGGSVDKLLVPRGSKLQ (sortase A motif: bold residues; a thrombin recognition sequence: underlined residues) was cloned into the Sac I and Xho I sites of the /D∆H_C_ plasmid to yield a construct encoding /D∆H_C_-CS and a C-terminally fused His_6_ tag. A synthetic gene encoding Gly_5_-IL-1β was cloned into the pET32b vector immediately after the enterokinase recognition site. The yielded constructs and pET30-heptamutant Sortase A (plasmid number 51141; Addgene) were separately transformed into *E. coli* strain BL21 DE3, and proteins were expressed using an auto-induction medium following the established protocol [64]. Briefly, bacteria overnight grown in Luria-Bertani broth medium were inoculated (1:1000 *v*/*v*) into ZYP-5052 auto-induction medium and cultured at 37 °C for ≈5 h with agitation (220 rpm/min). Bacteria were further cultured at 22 °C for ≈20 h with continuous shaking before harvesting at 4000× *g* for 40 min. Bacterial pellets from 1 L of culture were re-suspended in 40 mL of lysis buffer (20 mM HEPES, 145 mM NaCl, pH 8.0). After adding lysozyme (to a final concentration of 2 mg/mL), 800 units of benzonase nuclease (Millipore, Ireland) and a protease inhibitor cocktail III (1:200 *v*/*v*, Millipore, Ireland), *E. coli* cells were lysed at 4 °C for 1 h. After one freeze-thaw cycle, the lysate was centrifuged at 18,000× *g* for 1 h to remove debris. Recombinant proteins were then purified by IMAC, using Talon superflow metal affinity resin following the provided manual. The purified proteins were buffer exchanged into sample storage buffer (20 mM Hepes, 145 mM NaCl, pH 7.4) using PD-10 columns. The resultant /D∆H_C_-CS SC was either stored at −20 °C or nicked into DC, by thrombin (1 U of thrombin/mg of protein for 2 h at 22 °C), before the addition of phenylmethylsulfonyl fluoride (final concentration 1 mM). The concentration of nicked /D∆H_C_-CS was measured using Bradford reagent. Only the nicked /D∆H_C_-CS DC was used in the conjugation reaction. IMAC purified fusion protein Trx-H_6_-IL-1β was incubated with enterokinase in ratio 1 to 6.25 × 10^5^ for 3 h at 22 °C to separate the Trx-His_6_ tag from Gly_5_-IL-1β.

### 4.4. Conjugation of /D∆H_C_-CS with IL-1β or Synthesized Peptide

/D∆H_C_-CS (10 µM final concentration) and sortase A (25 µM, final concentration) were mixed with an enterokinase-cleaved Trx-His_6_ and Gly_5_-IL-1β mixture (0.3 mM, final concentration) in reaction buffer (50 mM Tris, 150 mM NaCl, 10 mM CaCl_2_, pH 7.4) at 37 °C for the times indicated. To purify the /DIL-1β conjugated protein, the reaction samples were buffer-exchanged into 50 mM Tris buffer (pH 8.0) and loaded onto a Resource Q column following a previously published protocol [29]. Briefly, after wash with Tris buffer with 30 mM NaCl, a stepwise gradient up to 1M NaCl was used to elute the conjugated protein and other contaminants.

To conjugate /D∆H_C_-CS to Gly_3_-CGRP_8-37_ or Gly_3_-substance P, /D∆H_C_-CS (10 µM, final concentration) and sortase A (25 µM, final concentration) were mixed with peptides (0.3 mM, final concentration) at 37 °C for 30 min. The reaction mixture was buffer-exchanged into the sample storage buffer using Zeba Spin Desalting Columns to stop the reaction and remove the excess peptides.

### 4.5. Measuring the Protease Activities of /DΔH_C_ Variants and the Bio-Activity of IL-1β Ligand

The proteolytic activities of /DIL-1β, /D-CGRP_8–37_ and control protein were assessed in vitro using a recombinant GFP-VAMP_(2–94)_-His_6_ substrate cleavage assay [65]. Briefly, the proteins were diluted to 100 nM in protease assay buffer HBS-20 (20 mM HEPES, 100 mM NaCl (pH 7.4), 10 µg/mL bovine serum albumin (BSA), 5 mM DTT and 10 µM ZnCl_2_) and incubated at 37 °C for 30 min to activate proteases. The proteins (100 nM) were serially diluted (2-fold) to a final concentration of 1.56 nM in HBS-20 buffer then mixed with equal volume of 1 mg/mL of GFP-VAMP_(2–94)_-His_6_ substrates. Following additional 30 min incubation at 37 °C, the reactions were stopped by adding equal reaction volume of ice-cold 2 × LDS sample buffer. The samples were analyzed by running on 12% Tris-glycine SDS-PAGE gels and stained by Coomassie solution.

The bio-activity of purified /DIL-1β was measured by the cell proliferation assay. RAW 264.7 cells were plated in a 96 well plate with ≈0.4 × 10^4^ cells/well, and cultured in Dulbecco’s modified Eagle’s medium (DMEM) containing 10% (*v*/*v*) heat-inactivated fetal bovine serum (FBS) and 1% (*v*/*v*) penicillin streptomycin, for 24 h at 37 °C, with 5% CO_2_. After 24 h incubation, a range of two-fold serial dilutions of commercial human IL-1β (from 500 pg/mL to 0.98 pg/mL), serial diluted /DIL-1β or /D∆H_C_-CS were added to the plate and incubated for a further 48 h at 37 °C, with 5% CO_2_. Treated cells were labelled with 10 μM of EdU for 16 h before fixation, which was followed by Click-iT^®^ EdU microplate assaying, according to the manual. Finally, the fluorescence, excitation/emission maxima 490/530 or 530/590 nm, was read using a plate reader. The percentage of bio-activity of the test proteins was calculated relative to the commercial human IL-1β using the standard curve based on data obtained using the human IL-1β.

### 4.6. Isolation and Culturing Primary Mouse Peritoneal Macrophages and Rat DRGs

Mouse peritoneal macrophages were isolated from the peritoneal cavity of around 8 week old CD1 mice and cultured in vitro, according to a protocol published by Zhang et al. [66]. Briefly, the skin at the front of the peritoneum was separated and removed from the peritoneum. A total of 5 mL of chilled Dulbecco’s phosphate-buffered saline (DPBS) was injected inter-peritoneally, and then fluid was collected from the peritoneal cavity. The collected fluid was centrifuged at 120× *g* for 8 min. The cell pellet was resuspended in a culture medium (DMEM/Nutrient F12 ham, 10% (*v*/*v*) FBS, 1% (*v*/*v*) penicillin streptomycin) and seeded ≈4 × 10^5^ cells per well into a 24 well plate. After 1 h incubation at 37 °C, with 5% CO_2_, the cells were washed with warmed DPBS three times, fresh culture medium was added and it was incubated for a further 24 h. More than 90% of cells should be macrophages [66].

rDRGs were isolated and cultured, following the published protocols [67,68]. Briefly, the dorsal root ganglion dissected from Sprague Dawley post-natal 0–3 rats were centrifuged for 1 min at 120 g. The isolated DRGs were then incubated at 37 °C for 30 min with DMEM medium, containing 2.4 U/mL dispase II and 5 mg/mL collagenase I. Following centrifugation for 5 min at 120 g, pellets were resuspended in DMEM medium with gentle trituration until cloudy. After centrifugation for 5 min at 120 g, cell pellets were resuspended in culture medium (DMEM medium containing 5% (*v*/*v*) FBS, 1% (*v*/*v*) penicillin/streptomycin, 50 ng/mL 2.5S NGF and 1 × B-27 supplement), and then ≈5 × 10^5^ cells/well were seeded in 48 well plates which were pre-coated with poly-l-lysine (0.1 mg/mL) and laminin (20 μg/mL). From day one onwards, cytosine-β-d-arabinofuranoside (Ara-C) (10 μM) was added into the culture medium, which was replaced every other day.

### 4.7. Treatment of Macrophages with/DIL-1β or/DΔH_C_-CS, Measuring VAMP3 Cleavage and IL-6 Release

After seeding the RAW cells (≈1 × 10^5^ cells per well) or primary mouse macrophages (≈4 × 10^5^ cells per well) in a 24 well plate for 24 h at 37 °C, 5% CO_2_, the cells were incubated with a range of two-fold serial dilutions of DIL-1β or control protein for 6 h at 37 °C, with 5% CO_2_. After removal of the medium, pre-treated macrophages were incubated with 0.3 mL stimulation solution (DMEM medium (RAW cells) or DMEM/Nutrient F12 ham (primary mouse macrophages), containing 100 ng/mL LPS, 500 pg/mL IFNγ and 1% penicillin/streptomycin) for 42 h at 37 °C, with 5% CO_2_ [49]. Then the supernatants were collected, and IL-6 concentration was analyzed by ELISA following instructions for the kit. Treated macrophages were lysed in 70 μL of 2 × LDS sample buffer, heated for 10 min at 95 °C and run on Bolt 12% Bis-Tris gels. Samples were transferred onto a PVDF membrane and probed with rabbit anti-SNAP23 (1:1000; 111 202; Synaptic Systems) and anti-VAMP1/2/3 (1:1000; 104 102; Synaptic Systems) antibodies overnight at 4 °C, followed by incubation with HRP donkey anti-rabbit secondary antibodies for 1 h at room temperature. Finally, blots were developed by ECL reagents and visualized using the G BOX Chemi-16 gel documentation system and intensities in each lane were quantified using Image J software [65].

### 4.8. Cell Viability Assay

The RAW cells were plated into a 96 well plate with ≈ 0.4 × 10^5^ cells/well and cultured for 24 h at 37 °C, with 5% CO_2_. On the following day, the cells were incubated with various concentrations of proteins for 44 h at 37 °C, with 5% CO_2_. Then each well was replaced with 110 μL culture medium, containing 10 μL alamar blue reagents, and further, incubated for 4 h, before reading absorbance at 570 nM by plate reader.

### 4.9. Analysis of VAMP1 Cleavage and SP Release in rDRGs

After 6–7 days in vitro, the rDRGs were incubated with various doses of /D-CGRP_8-37_ or /DΔH_C_-CS for 24 h at 37 °C, with 5% CO_2_. Before harvesting, treated rDRGs were incubated with 0.2 mL of K^+^ stimulation buffer (mM; 22.5 HEPES, 75 NaCl, 63.5 KCl, 1 MgCl_2_, 2.5 CaCl_2_, 3.3 glucose and 0.1% (*w*/*v*) BSA, pH 7.4) for 30 min at 37 °C, with 5% CO_2_ [68]. The basal release was measured by incubation of other spare cells with 3.5 mM low potassium (LK) buffer with adjusting of NaCl concentration to reach isotonic balance [68]. The supernatants were then collected and used for SP EIA analysis, according to instructions provided with the kit. Treated rat DRGs were lysed in 60 μL 2 × LDS sample buffer and analyzed by Western blotting using a rabbit antibody which recognizes intact VAMP1 but not BoNT/D in cleaved form (1:1000; 104 002; Synaptic Systems). VAMP1 cleavage was normalized according to a loading control (syntaxin 1) before analysis relative to the toxin free control cells.

### 4.10. Statistical Analysis

All the raw data were analyzed using Microsoft Excel 2010. Graph Pad Prism 4.0 was used to plot the calculated data points and perform the statistical analysis. The number of independent experiments (*n* value) represented in a graph is highlighted in the corresponding figure legends. The significance between treatments was evaluated using the student’s unpaired, two-tailed *t*-test.

## Supplementary Materials

The following are available online at https://www.mdpi.com/1422-0067/21/1/262/s1.

## Abbreviations

AEX	Anion-exchange chromatography
Ara-C	Cytosine-β-D-arabinofuranoside
BoNT	Botulinum neurotoxin
BoNT/D	Botulinum neurotoxin D
BOTOX	Botulinum toxin A complex
CGRP	Calcitonin gene-related peptide
CGRP_8-37_	CGRP receptor antagonist
CS	C-terminal sortase motif
DC	Di-chain
/DΔH_C_	BoNT/D lacking the neuronal binding domain H_C_
/D-CGRP_8-37_	/DΔH_C_-CS and CGRP_8-37_ ligated conjugate
/DIL-1β	/DΔH_C_-CS and IL- 1β ligated conjugate
DMEM	Dulbecco’s modified Eagle’s medium
DPBS	Dulbecco’s phosphate-buffered saline
DRGs	Dorsal root ganglion neurons
DTT	Dithiothreitol
ECL	Enhanced chemiluminescence reagent
EIA	Enzyme immunoassay
FBS	Fetal bovine serum
H_6_	His6 tag
HC	Heavy chain domain
H_C_	C-terminal half of H_C_
HK	High potassium
H_N_	N-terminal half of H_C_
IFNγ	Interferon gamma
IL-1β	Interleukin 1β
LC	Light chain domain
LK	Low potassium
LPS	Lipopolysaccharides
NGF-2.5S	Nerve growth factor 2.5S
NSAIDs	Nonsteroidal anti-inflammatory drugs
RA	Rheumatoid arthritis
SC	Single-chain
SNAP-25	Synaptosomal-associated protein 25k
SNAREs	Soluble *N*-ethylmaleimide-sensitive factor attachment protein receptors
TNFα	Tumor necrosis factor-α
Trx	Thioredoxin tag
Trx-H_6_	Trx-His_6_ tag
Trx-H_6_-IL-1β	Thioredoxin-His_6_-Gly_5_-IL-1β protein
US	Unstimulated
VAMP	Vesicle associated membrane protein
