# Supplementary material for: New Engineered-Botulinum Toxins Inhibit the Release of Pain-Related Mediators
Source: Int J Mol Sci. 2019 Dec 30;21(1):262. doi: 10.3390/ijms21010262 (PMC6981458; doi:10.3390/ijms21010262)
Supplement: Supplementary file 1 [file ijms-21-00262-s001.pdf]

Supplementary information

New engineered-botulinum toxins inhibit the release of pain-related mediators

Minhong Tang, Jianghui Meng and Jiafu Wang

School of Biotechnology, Faculty of Science and Health, Dublin City University, Collins Avenue, Glasnevin, Dublin 9, Ireland.

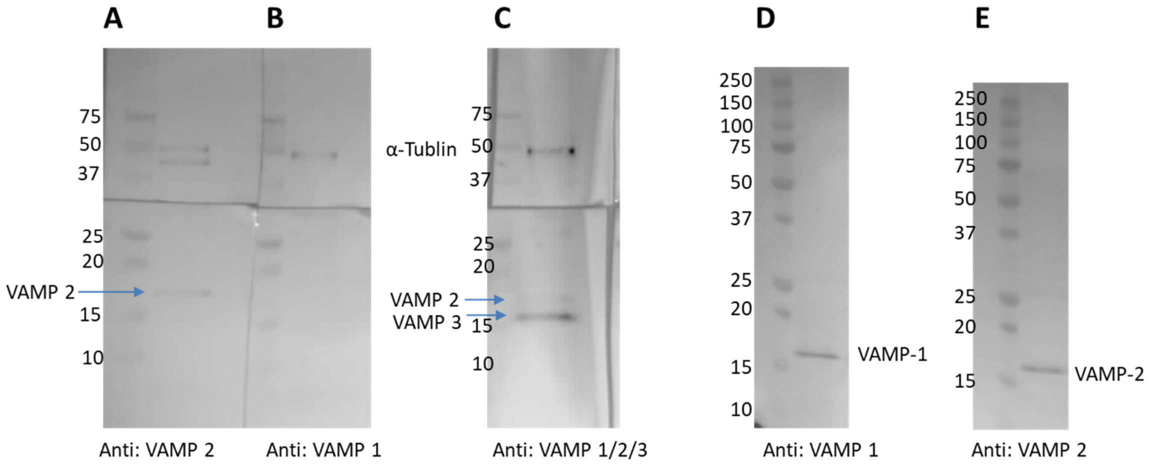

Figure S1. RAW 264.7 cells express predominately VAMP3 isoform. (**A-C**) Cell lysates from cultured RAW cells were subjected to SDS-PAGE followed by Western blotting using indicated antibodies against VAMP1, VAMP2, VAMP1/2/3 or α-Tublin. Note that, samples were incubated with VAMP antibodies for 20 h at 4°C whereas anti α-Tublin antibody was incubated with samples for 1 h at 22°C. Western blots show RAW cells express predominately VAMP3 isoform. Anti-VAMP2 antibody detected a faint band in WB, corresponding to predicted size of VAMP 2 (~17 kDa). VAMP1 was not detected under the same condition. In contrast, VAMP 1 (**D**) and VAMP 2. (**E**) in cell lysates from cultured mDRGs were easily detected in WB.

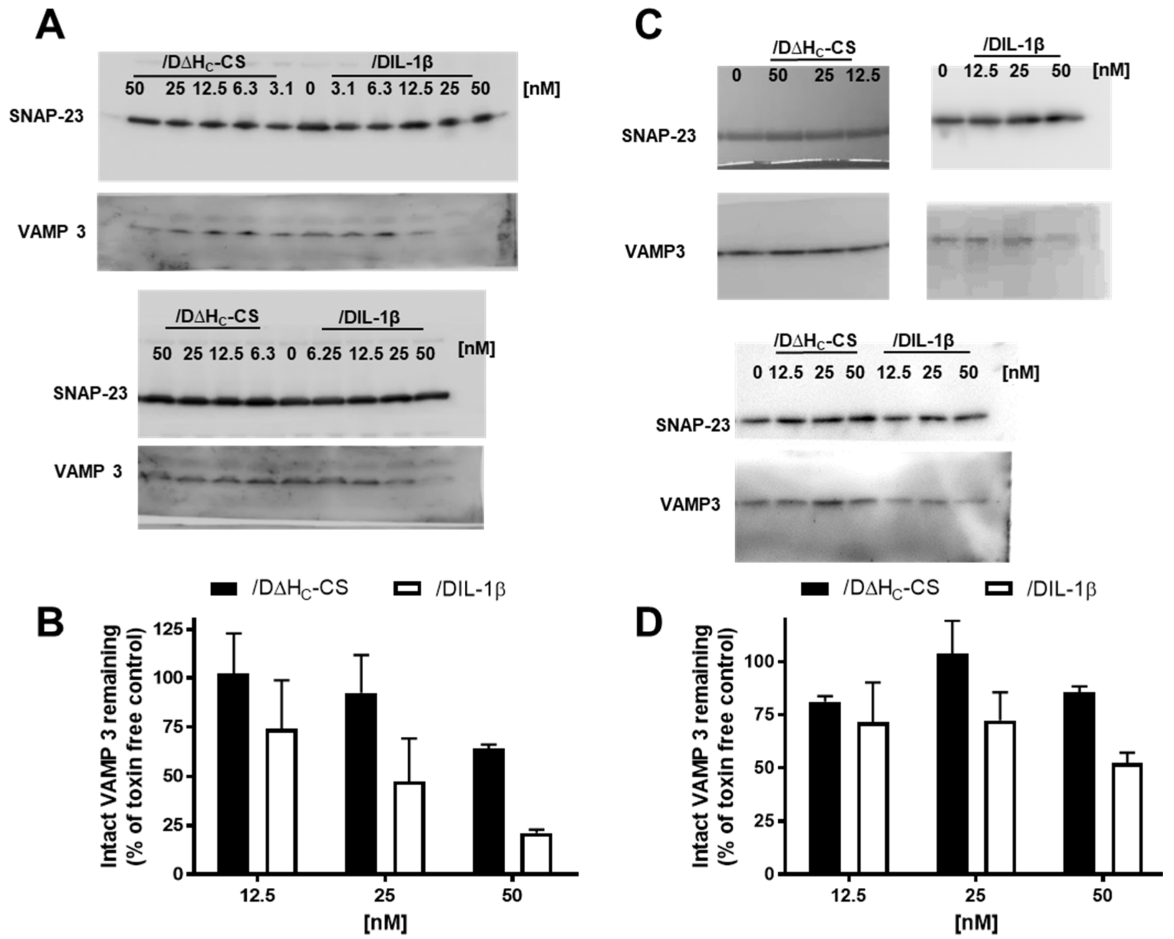

Figure S2. Effect of /DIL-1β and /DΔH_C-CS on cleavage of VAMP 3 in cultured macrophages. Cultured RAW264.7 cells (A, B) and primary mouse macrophage cells (C, D) were treated with /DIL-1β or /DΔH_C-CS for 6 h before stimulation with LPS and IFNγ for 42 h. Cells were dissolved in LDS sample and cell lysates were subjected to SDS-PAGE followed by Western blotting (A, C) using an antibody against VAMP1/2/3. Detection of VAMP3 proved to be more difficult than SNAP-23. (B, D) Plots showing the differential cleavage of VAMP 3 by /DIL-1β and /DΔH_C-CS. VAMP 3 cleavage was normalized according to a loading control (SNAP-23) before analysis relative to the toxin free control cells. Data plotted are mean ± S.E.M.

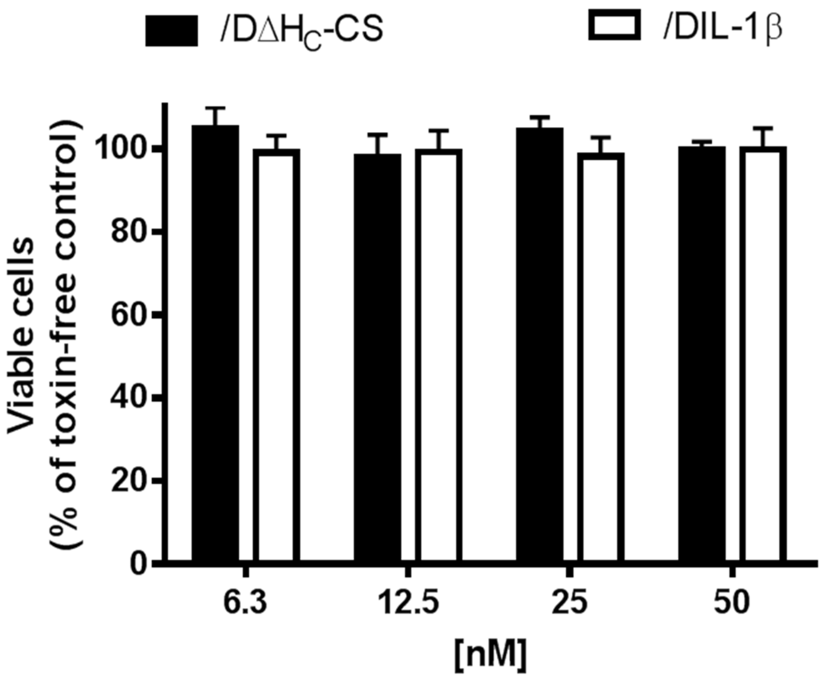

Figure S3. Effect of /DIL-1β and /DΔHc-CS on RAW 264.7 cell viability. RAW cells were plated into a 96 well plate with ~ 0.4×10^5 cells/well and cultured for 24 h at 37°C, 5% CO_2. On the following day, the cells were incubated with various doses of /DIL-1β or /DΔH_C-CS for 44 h, followed by 4 h incubation with alamar blue contained culture medium before reading at absorbance 570 nm. Viable cells after treatment was quantified as a % of the toxin free control sample (vehicle) treated cells. Data graphed are mean ± S.E.M. from 2 independent experiments.

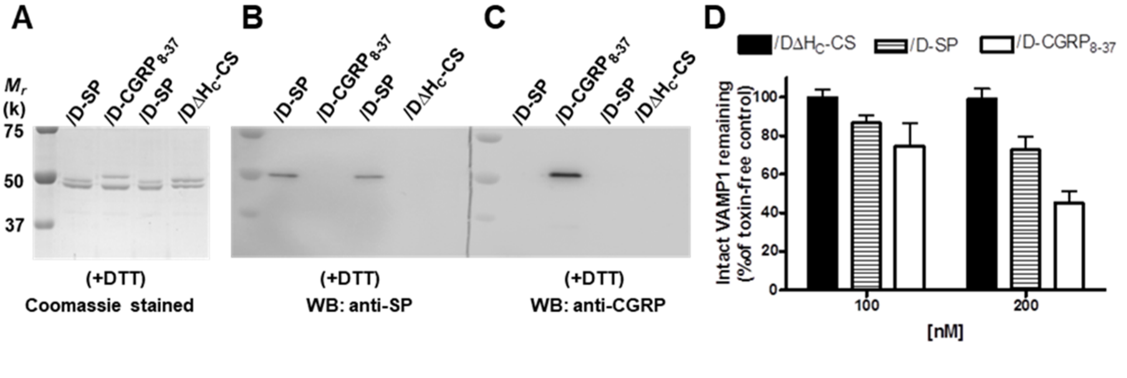

Figure S4. Effect of /D-SP conjugate on VAMP 1 cleavage in cultured DRGs. /D-SP conjugate was produced as for /D-$CGRP_{8\text{-}37}$. DTT reduced conjugate samples were subjected to SDS-PAGE followed by Coomassie staining (**A**) or Western blotting using an antibody against substance P (**B**) or an antibody against CGRP (**C**). (**D**) Rat DRGs were incubated with /D-SP conjugate for 24 h at 37°C. The cells were then

harvested in LDS-sample buffer for Western blotting. Intact VAMP1 remaining after overnight treatment was plotted as % of toxin-free control. SP: substance P. Data are mean ± SEM from two independent experiments. Data for /D-$CGRP_{8\text{-}37}$ conjugate and control protein from Fig. 5 were replotted here for comparison.
